# 

**Published:** 1892-02

**Authors:** 


### CONTENTS.

ORIGINAL COMMUNICATIONS—

ABDOMINAL SECTION IN A CASE OF CYST OF THE MESENTERY, WITH REMARKS. By J. Adrian Goggans, M. D., Alexander City, Ala...... 705

CLINIC REPORTS. By W. F. Westmoreland, M. D., Atlanta Medical College........ 711

REPORT OF CLINIC OF A. W. CALHOUN, M. D. By Jas. H. Latimer, Jr. 714

REPORT OF PERINEAL SECTION. By F. W. McRae, M. D.......... 717

SOME REMARKS CONCERNING THE USE OF MERCURY IN THE TREATMENT OF SYPHILIS. By Dr. F. A. Rietema, Rotterdam.......... 718

THE ASEPTIC CLOSURE OF LONG STANDING SINUSES HAVING THEIR ORIGIN IN TUBERCULAR JOINTS. Reprint by H. Augustus Wilson, M. D., Philadelphia.......... 725

SOCIETY REPORTS—

THE CLINICAL SOCIETY OF MARYLAND.......... 733

CORRESPONDENCE—

OUR NEW YORK LETTERS.......... 736, 743

EDITORIAL—

SPECIAL NOTICE.......... 750

THE OPEN DOOR FOR QUACKERY.......... 750

THE INFLUENZA AGAIN.......... 753

SELECTIONS.......... 755-764

MEDICAL ITEMS.......... 765

BOOK REVIEWS.......... 767

INDEX TO ADVERTISEMENTS.......... xiii

ITEMS OF INTEREST.......... xi and xii

CLUB RATE PAGE.......... xxiv

